# Rates of thyroid malignancy by FNA diagnostic category

**DOI:** 10.1186/1916-0216-42-61

**Published:** 2013-12-20

**Authors:** Blair A Williams, Martin J Bullock, Jonathan R Trites, S Mark Taylor, Robert D Hart

**Affiliations:** 1 Department of Surgery, Division of Otolaryngology – Head & Neck Surgery, Dalhousie University, Halifax, NS, Canada; 2 Department of Pathology, Dalhousie University, Halifax, NS, Canada

**Keywords:** Fine needle aspiration, FNA, Thyroid cancer

## Abstract

**Background:**

Fine needle aspiration (FNA) of thyroid nodules is a cornerstone of surgical decision making in thyroid cancer. The most widely utilized system for reporting thyroid FNA results is the Bethesda System, which includes predicted malignancy rates for each FNA category. To date there have been few studies to determine whether these predictions are widely applicable.

**Methods:**

All thyroid FNA results at the Queen Elizabeth II Health Science Centre from 2006–2010 were included in this study. The results were tabulated by FNA category and the health records were reviewed to determine whether the patient went on to have surgery and the result of surgical histopathology. Rates of malignancy were calculated and compared to published values.

**Results:**

A total of 1491 thyroid FNAs were included in the study, representing 1117 individual patients with available health records. The majority of these FNAs were Benign, but the proportion of Unsatisfactory FNAs was higher than predicted while Malignant and Suspicious for Malignancy were lower than predicted. Surgery was performed on 388 patients and 110 were positive for malignancy (28%). The malignancy rate for each FNA category was higher than predicted based on literature values.

**Conclusions:**

The proportions of FNA diagnoses and the rates of malignancy for each FNA category at our institution were not consistent with predicted values. It is important for clinicians to base their surgical recommendations on institution specific malignancy rates, not solely on literature values.

## Background

Thyroid cancer has the most rapidly increasing incidence of all major cancers in Canada [1]. Part of this increased incidence is attributable to earlier detection of thyroid nodules with improved diagnostic imaging techniques. The majority of thyroid nodules are benign but all warrant investigation to rule out malignancy. Assessment of thyroid nodules is a common reason for referral to an otolaryngologist. Early detection and treatment of malignant nodules is associated with excellent outcomes [1]. Currently, treatment decisions for thyroid nodules rely heavily on the results of fine needle aspiration (FNA) of the thyroid.

Fine needle aspiration is an important diagnostic tool in the evaluation of thyroid nodules. This cytological test is widely available and easy to perform in an outpatient setting. The result of an FNA is a cornerstone in determining whether to perform surgery and which type of surgery to offer (i.e., hemithyroidectomy or total thyroidectomy). A key factor in the application of FNA results is consistency among reports. As interpretation of thyroid FNAs evolved, a number of systems were developed to categorize the results. The most recent and most widely used approach is the Bethesda System. With the release of the Bethesda guidelines, there now exists a standard schema for the classification of thyroid cytologic specimens [2]. This has been a great advance in the treatment of thyroid cancer.

The Bethesda System defines 6 categories: *non-diagnostic*, *benign*, *atypia of undetermined significance* (*AUS*), *suspicious for a follicular neoplasm* (*SFN*), *suspicious for malignancy* (*SFM*), and *malignant*. The Bethesda system is not just a classification system but also goes into great detail about cytological criteria for categorizing the findings on each FNA. The system also details the risk of malignancy for each category, based on large patient series, as well as the suggested management for each FNA result. Though this system has been widely adopted, there has been little research to audit whether the actual risks of malignancy are consistent with those reported. There have been no Canadian studies to determine whether the published risks of malignancy are applicable in Canada. Despite this, patients are often counseled and operative recommendations made based on Bethesda data.

Given the pivotal role played by FNA in the work-up of a thyroid nodule, it is important to ensure that this tool is providing reliable data. There have been a small number of American studies that compared the results of the preoperative thyroid FNA to the final histology after surgical excision of the gland [3-5]. This provides an estimate of the accuracy of the FNA. These studies have largely supported the clinical utility of FNAs but broad application of their results is difficult given differences among centers and systemic differences between Canada and the United States.

We undertook an audit of our thyroid FNA results to determine whether our results were consistent with the Bethesda System and with literature values from large series in the literature. Our goals were to provide quality assurance by describing the various proportions of diagnoses made and to improve our surgical decision-making by having an accurate picture of malignancy risks at our institution. The setting is a tertiary care center in Canada that is representative of other such centers in Canada.

## Methods

Institutional research ethics board approval was obtained. All patients who received FNA of the thyroids between 2006 and 2010 were included in our database. Investigation of thyroid nodules at our institution follows established guidelines from the American Thyroid Association [6]. The results of their FNA, whether or not they went on to have surgery, and the results on surgical pathology were collected. The cytopathological system used at our institution was slightly different from Bethesda in that is used 5 diagnostic categories, grouping *AUS* and *SFN* together into *Abnormal*. We were able to extrapolate the *SFN* results based on pathologist comments to arrive at six categories that corresponded to the six in the Bethesda System and in other published studies. Only comments that included the phrases “Diagnosis favors neoplasm” or “Suspicious for neoplasm” were categorized as *SFN*. Initially 346 FNAs were categorized as *Abnormal.* Sixty-five were subsequently extrapolated to *SFN* (Table 1).

**Table 1:** The distribution of thyroid FNAs by diagnostic category, with percentage in parentheses

**FNA result**	**Number of FNAs (%)**
Unsatisfactory	431 (28.9)
Benign	681 (45.7)
Abnormal	281 (18.8)
SFN	65 (4.4)
SFM	19 (1.3)
Malignant	14 (0.9)

FNA – fine needle aspiration, SFN – Suspicious for Neoplasm, SFM – Suspicious for Malignancy.

A number of the surgical specimens contained papillary thyroid microcarcinoma (i.e., a focus less than 1 cm in greatest diameter). These were categorized as benign if there was no macroscopic cancer present because the sub-centimeter foci would not be targeted by FNA. In analyzing the malignancy rates, if an individual patient received multiple FNAs, the most worrisome FNA result was used when comparing to surgical pathology.

Our two major outcomes were 1) what proportion of all thyroid FNAs does each category represent and 2) for patients who go on to surgery, the rate of malignancy within each FNA category.

Our results were tabulated in Microsoft Excel and percentages calculated. The results were compared to the Bethesda System and to other large studies. The Bethesda System gives ranges for risk of malignancy. We used the midpoint of the range for our graphic comparisons.

## Results

Between April 2006 and December 2010, there were 1491 thyroid FNAs interpreted at our centre. The distribution of FNA results by category is reported in Table 1.

There were 1167 individual patients represented in the above values, as some had repeat FNAs. Of these patients, 1117 had health records of their surgery available, 915 female and 202 male. Of these patients, 388 (38.5%) went on to have thyroid surgery: 246 hemithyroidectomies, 127 total thyroidectomies, 13 completion thyroidectomies, and 2 open biopsies. Of those patients who received surgery 110 had malignancy, for an overall malignancy rate of 28.0%. The specific diagnoses were as follows: 87.3% papillary thyroid cancer, 4.6% follicular thyroid cancer, 2.7% medullary thyroid cancer, 2.7% renal cell carcinoma metastases, 1.8% lymphoma, and 0.9% poorly differentiated thyroid cancer. The correlation of FNA category to surgical pathology for these patients is reported in Table 2.

**Table 2:** Correlation of FNA category to surgical pathology and overall malignancy rate for each category

**FNA category**	**Surgical pathology (%)**				**Malignancy rate (%)**
	**PTC**	**FTC**	**MTC**	**Other**	
Unsatisfactory	14.5	0.0	0.0	3.6	18.2
Benign	14.9	0.0	0.0	1.1	16.0
Abnormal	22.2	1.2	0.6	0.6	24.7
SFN	23.9	6.5	0.0	2.2	32.6
SFM	76.5	0.0	11.8	5.9	94.1
Malignant	100.0	0.0	0.0	0.0	100.0

Values are expressed as percentages of all FNAs in each category. FNA – fine needle aspiration, SFN – Suspicious for Neoplasm, SFM – Suspicious for Malignancy.

## Discussion

This study presents a thorough audit of thyroid FNA and associated malignancy rates at a Canadian tertiary care canter. Our findings suggest that the distribution of FNA diagnoses is not consistent with large American patient series and the malignancy rates are not consistent with the Bethesda System. This suggests that when making treatment recommendations and counseling patients, surgeons should use data from their own institution in addition to published values.

A 2007 study from Yang et al. correlated FNA results with surgical histology in series of 4703 patients from two institutions [3]. In this series, 1072 patients went on to have surgery and the results from those patients were used to estimate malignancy rates. Overall, they found that thyroid FNA using the Bethesda System to be accurate and clinically useful in surgical decision-making. Subsequently, Jo and colleagues published their findings from a series of 3080 thyroid FNAs [4]. In this study, 892 cases had post-operative histology available for comparison. Their results were very similar to those of Yang and colleagues. More recent research compared FNA results to histology in a community practice [5]. The results of 1382 thyroid FNAs were summarized along with the malignancy rates by FNA category for 221 patients who went on to have surgery. The overall results of this study differed slightly from the larger studies, possibly due to different practices and resources available at a community centre. The results of the three papers outlined above are summarized in Tables 3 and 4.

**Table 3:** Comparison of FNA diagnoses between this study and the published values in other studies, expressed as a percentage of all FNAs

**FNA diagnosis**	**This study N = 1491**	**Yang et al., 2007**[3]**N = 4703**	**Jo et al., 2010**[4]**N = 3080**	**Wu et al., 2011**[5]**N = 1382**
Unsatisfactory	28.9	10.4	18.6	20.1
Benign	45.7	64.6	59.0	39.0
Abnormal	18.8	3.2	3.4	27.2
SFN	4.4	11.6	9.7	8.4
SFM	1.3	2.6	2.3	2.6
Malignant	0.9	7.6	7.0	2.7

FNA – fine needle aspiration, SFN – Suspicious for Neoplasm, SFM – Suspicious for Malignancy.

**Table 4:** Comparison of malignancy rates by FNA category among this study, the Bethesda System, and published values in other studies

**FNA category**	**This study n = 388**	**Bethesda range**	**Yang et al., 2007**[3]**n = 1052**	**Jo et al., 2010**[4]**n = 892**	**Wu, 2011**[5]**n = 221**
Unsatisfactory	18.2	1-4	10.9	8.9	14
Benign	16.0	0-3	7.3	6.5	3
Abnormal	24.7	5-15	13.5	17	6
SFN	32.6	15-30	32.2	25.4	22
SFM	94.1	60-75	64.7	70	56
Malignant	100.0	97-99	98.6	98.1	100
Overall	28.4	--	45.3	31.3	29.0

FNA – fine needle aspiration, SFN – Suspicious for Neoplasm, SFM – Suspicious for Malignancy.

A comparison of the distribution of FNA diagnoses in this study and the above studies is summarized in Table 3. Though the results are inconsistent among the published values, this study shows a proportion of *non-diagnostic* FNAs that is higher than all the other studies, and rates of *SFM* and *malignant* that are lower than all the other studies. The high number of *non-diagnostic* FNAs may reflect shortcoming in the specimen preservation and preparation and warrants further study within the institution. Many large centers in the United States have dedicated cytopathologists for immediate on-site evaluation of specimen adequacy and resampling if necessary. The difference could also be explained by sampling error. Recent studies have shown improved yield and decreased rate of *non-diagnostic* FNAs with routine use of ultrasound guidance of FNAs, compared to palpation guidance [7,8]. Ultrasound guided FNA of the thyroid may also be a more cost-effective approach [9].

The low proportion of *SFM* and *malignant* is more concerning. Though it may be due to a difference in the populations served by the current institution, it may also indicate that malignant diagnoses are being under-called. This would lead to delays in treatment or to hemi-thyroidectomies that should be total thyroidectomies. This concern is supported by the comparison of malignancy rates among published studies in Table 4. There is a higher malignancy rate in this study for each of the FNA categories when compared to both the Bethesda System and to other published studies. The *SFM* malignancy rate (94%) is almost as high as *malignant* (100%), potentially leading to hemi-thyroidectomies that should be total, according to the treatment guidelines from the Bethesda System.

The only follicular thyroid carcinomas encountered in this study were all classified as *abnormal* or *SFN* (Table 2). This is consistent with classic teaching that follicular neoplasms fall into an abnormal or atypical group since cytology cannot detect capsular or vascular invasion, differentiating adenoma from carcinoma. The majority of malignancies in the *abnormal* and *SFN* groups, however, were papillary thyroid carcinoma. Typically, PTC should be categorized as *SFM* or *Malignant.* These cases were reviewed specifically and all cases of PTC categorized as *abnormal* or *SFN* were follicular variant of PTC, a subtype that has long posed diagnostic challenges for cytopathologists.

Another interesting finding in the *abnormal* and the extrapolated *SFN* categories was the much higher malignancy rate in the *SFN* group: 25% versus 33%. The number of FTC lesions was also much higher in the *SFN* group: 1.2% versus 6.5% of cases. This indicates that at our centre an additional category would be useful in risk stratifying patients with indeterminate FNA results.

**Figure 1:**
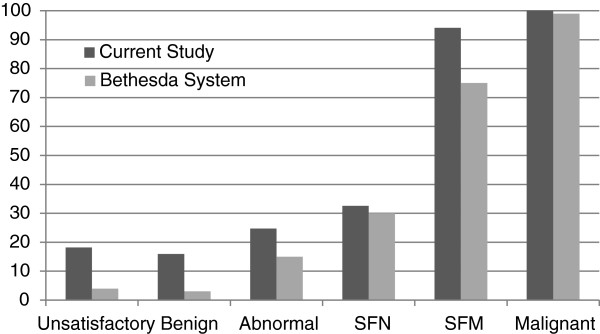
**Comparison of malignancy rates (in percentages) between the current study.** Bethesda System values are given as the midpoint from the reported range. FNA – fine needle aspiration, SFN – Suspicious for Neoplasm, SFM – Suspicious for Malignancy.

The malignancy rates are considerably higher in the present series for *Benign* and *Non-diagnostic* FNAs, compared to Bethesda (Figure 1). Though an interesting result, a major factor in this difference is attributable to selection bias. In the Bethesda System, they report the malignancy risk for all comers. For our study the malignancy rates were determined from those patients who underwent surgery. The majority of patients with *Benign* and *Non-diagnostic* FNAs did not receive surgery; only those with worrisome features clinically or on ultrasound.

Though this study provides much useful information, there are a number of inherent limitations. The system to categorize thyroid FNA results at our institution is not the same as the Bethesda System. Though we extrapolated analogous categories, it is not a perfect match. Furthermore, our extrapolated category likely under-represents the number of FNAs that would be categorized as *SFN* due to the high degree of variability in the detail in the pathologist comment section. As this is a retrospective study, multiple pathologists of various levels of experience reviewed the FNAs. Categorizing papillary thyroid microcarcinoma was also a dilemma in this study. Though it has clinical significance we elected to categorize an isolated focus as *benign*. The aim of this study was to evaluate the quality FNA as a diagnostic tool. A nodule less than 1 cm in diameter would not have been targeted by FNA during the data collection period of this study. Current American Thyroid Association guidelines recommend FNA of lesions 0.5 cm or larger if the patient is high risk. We did not have a full data set of risk factors for the patients in this study and so could not retrospectively assign risk categories [6].

## Conclusion

The rates of malignancy were considerably higher in this series than would be predicted by the Bethesda system and literature values. Extrapolation of other categorization systems into the Bethesda system will not necessarily provide rates of malignancy as predicted by Bethesda. Pathology departments need to make sure that when the Bethesda system is adopted, the specific criteria for each category are followed carefully. The difference in malignancy rates could have important implications in patient care and surgical decision-making. Though it may be tempting to use values from the ‘best fit’ literature schemes, this study underscores the importance of using malignancy rates specific to the institutional classification system and the population served.

## Competing interests

The authors declare that they have no competing interests.

## Authors’ contributions

BAW performed chart reviews, collected and organized the data, and prepared the manuscript. MJB built the database, provided conceptual direction and assisted in the interpretation of the results. JRT and SMT gave conceptual direction, and assisted in the interpretation of the results and the preparation of the manuscript. RDH was responsible for the synthesis of the research question, design of the project, interpretation of the results, and assisted in the preparation of the manuscript. All authors read and approved the final manuscript.
